# Macroalgae size refuge from herbivory promotes alternative stable states on coral reefs

**DOI:** 10.1371/journal.pone.0202273

**Published:** 2018-09-18

**Authors:** Cheryl J. Briggs, Thomas C. Adam, Sally J. Holbrook, Russell J. Schmitt

**Affiliations:** 1 Department of Ecology, Evolution and Marine Biology, University of California, Santa Barbara, CA, United States of America; 2 Marine Science Institute, University of California, Santa Barbara, CA, United States of America; University of California, UNITED STATES

## Abstract

Coral reef systems can undergo rapid transitions from coral-dominated to macroalgae-dominated states following disturbances, and models indicate that these may sometimes represent shifts between alternative stable states. While several mechanisms may lead to alternate stable states on coral reefs, only a few have been investigated theoretically. We explore a model that illustrates that reduced vulnerability of macroalgae to herbivory as macroalgae grow and mature could be an important mechanism: when macroalgae are palatable to herbivores as juveniles, but resistant as adults, coral-dominated and algae-dominated states are bistable across a wide range of parameter space. We compare two approaches to global sensitivity analysis to rank the relative importance of the model parameters in determining the presence and magnitude of alternative stable states, and find that the two most influential parameters are the death rate of coral and the rate of maturation of algae out of the vulnerable stage. The Random Forest approach for global sensitivity analysis, recently adopted by ecologists, provides a more efficient method for ranking the relative importance of parameters than a variance-based approach that has been used frequently by computer scientists and engineers. Our results suggest that managing reefs to reduce chronic stressors that cause coral mortality and/or enhance the growth rates of algae can help prevent reefs from becoming locked in a macroalgae-dominated state.

## Introduction

Ecological systems can switch rapidly between alternate states with dissimilar structure and rates of key ecosystem processes [1]. In many cases these shifts involve transition to a less desirable state with reduced capacity to provide important ecosystem services [2]. Abrupt state changes are frequently persistent (e.g., regime or phase shift), and may represent shifts to an alternative stable state [3]. When alternative stable states exist, it is critical to identify the mechanisms that can maintain a state change to inform managers of the specific feedbacks that need to be broken to restore the system [4,5].

On coral reefs, loss of herbivores to overfishing can lead to phase shifts from coral- to algae-dominated systems, compromising the capacity to build reefs and provide habitat for other organisms [6,7]. While several models have demonstrated that such shifts could represent alternative stable states [8], there has been skepticism about whether they capture the important attributes of the systems [9] and/or whether most coral reefs exist in parameter space where alternative stable states are even possible [10]. In models with alternative stable states, a small change in a biological process may lead to a rapid shift to a less desirable state, but a much larger change in that same biological process may be required to restore the system to its original state (a phenomenon called “hysteresis”). For example, on a coral-dominated reef, a small decrease in the intensity of herbivory may lead the system to shift rapidly to an algae-dominated state. If the system has alternative stable states, a much larger increase in the intensity of herbivory may be necessary for the coral-dominated state to be restored. The primary mechanism leading to alternative stable states and hysteresis in most models of coral reef systems is the dilution of grazing intensity as corals decline and substrate suitable for the establishment and growth of algae increases [11,12] (but see [13]). However, herbivores frequently increase in abundance following coral decline, thereby maintaining high grazing intensity even as the area of substrate that can be grazed increases [14–17]. Several additional feedbacks have been proposed that could contribute to alternative stable states on coral reefs [5,18], but these have received less theoretical attention (but see [19]).

One feedback that has strong empirical support is the resistance of macroalgae to herbivores once they reach a size refuge [20]. Size refuges from herbivory are common among primary producers in terrestrial and marine systems, and may occur due to the onset of chemical and physical defenses as plants develop [21,22]. In addition, on coral reefs, the major herbivores lack the digestive machinery required to break down the refractory compounds present in large seaweeds, and feed instead on small algal turfs (multi-species assemblages of filamentous algae, macroalgal spores, microalgae, and detritus) [23]. Thus, macroalgae may achieve a size refuge due to the acquisition of structural and/or chemical defenses that make them less palatable to herbivores, and/or because of an increased ability of herbivores to detect and avoid feeding on large individuals. For example, the tropical brown alga, *Turbinaria ornata*, a dominant macroalga on Pacific coral reefs, becomes resistant to herbivory once it reaches approximately 2 cm in height [24].

Here we model how stage-structure in the susceptibility of macroalgae to herbivores influences the dynamics of coral-algae phase shifts and the likelihood of alternative stable states. While most simple models of species interactions assume that all individuals within a species are identical (and therefore can be described by a single state variable), “stage-structured” models allow for changes in the demographic rates (e.g., survival, growth, and fecundity) of the organisms within a species as they age, grow, and develop. We compare the results of a model with an unstructured macroalgae population to a model in which only young/small macroalgae are vulnerable to herbivory, while old/large algae are invulnerable.

In addition, we compare various approaches to sensitivity analysis to identify regions of parameter space where this mechanism is likely to be most important. Models are necessarily simplified depictions of complex ecological systems, and their parameters are often idealized representations of biological processes. Because it is difficult to obtain exact values of many model parameters, sensitivity analysis allows determination of the degree to which our conclusions depend on choice of parameter values. Local sensitivity analysis (LSA), in which parameter values are varied one at a time from a default set, requires that this “default” parameter set is defined, and cannot capture the effects of interactions between parameters. Global sensitivity analysis (GSA), in contrast, investigates the effects of variation in the values of all parameters simultaneously across their entire feasible range. There are, however, different approaches for GSA and there is no clear consensus on which approach is most informative for a given circumstance [25]. Here we contrast the results of local sensitivity analysis with two approaches for GSA: a Random Forest approach that has been used recently in the ecological literature [26], and a much more computationally intense variance-based approach that has been used frequently by computer scientists and engineers [27,28].

## Materials and methods

### Description of models

We developed two models of the temporal dynamics of the fraction of space on a coral reef occupied by key classes of benthic space holders: coral, macroalgae, and turf algae. In nature, each of these classes may include a diverse assemblage of species, but we follow the common simplifying modeling assumption that the dynamics of each class can be represented by a single (or two) state variable(s). We assume that any free space on the reef is immediately covered with turf (low-growing filamentous algae <10 mm in height), and therefore in the models, “free space” is synonymous with “turf”. In the model, crustose coralline algae are also included in the “turf” category, because these substrates (turf, crustose coralline algae) are collectively able to be overgrown by either coral or macroalgae. This assumption of the model is, however, an oversimplification–on real coral reefs crustose coralline algae are resistant to grazing, while turf algae rapidly regrow after being grazed. We assume that macroalgae, which compete with corals for space, have the potential to be controlled by herbivorous fish. We do not explicitly model the fish population dynamics, nor make the assumption common to many other coral reef models [11] that the grazing pressure on macroalgae exerted by fish increases as the availability of macroalgae decreases (as might occur if fish density remains constant). Instead, we include the loss of algae due to herbivory in the macroalgae death rate, and explore the impacts of altering this parameter on the outcome of the coral-macroalgae interaction.

#### Unstructured macroalgae model (all macroalgae are equally vulnerable to herbivory)

In our first model, we include macroalgae as a single, unstructured, state variable, such that all life stages are equally vulnerable to herbivory. This model describes the dynamics of the fractions of benthic space on the reef occupied by coral (*C*), macroalgae (*M*), and turf (or free space, *T*). At any point in time, all space on the reef is in one of these three possible states, such that *C + M + T = 1*. The equations describing the temporal dynamics are:

$$\frac{dC}{dt}=\phi_{C}T+g_{TC}TC-{\gamma g}_{TM}MC-d_{C}C$$ (1)

$$\frac{dM}{dt}=\phi_{M}T+g_{TM}TM+{\gamma g}_{TM}MC-d_{V}M$$ (2)

with *T = 1 –C–M*. We assume that both coral and macroalgae can recruit either locally or from outside of the local system (open recruitment). Open recruitment of propagules occurs only to free space for both corals (at rate *ϕ*_*C*_) and macroalgae (at rate *ϕ*_*M*_). Lateral growth of coral can occur only into free space, but macroalgae can grow over either free space or coral. *g*_*TC*_ is the rate at which free space is taken up due to the combination of local recruitment of coral to free space and the lateral growth of coral over free space. *g*_*TM*_ is the combined rate of local recruitment and lateral growth of macroalgae over free space. We assume that the growth rate of macroalgae is usually slower over coral than over free space, so the rate of growth of macroalgae over coral is γ*g_*TM*_ (with scaling constant *γ* ≤ 1). *d*_*C*_ and *d*_*V*_ are the death rates of coral and vulnerable macroalgae (which in this first model includes all macroalgae), respectively. Because our models do not include herbivorous fish as a dynamic variable, the grazing pressure on macroalgae by fish is included in the macroalgae mortality rate (*d*_*V*_). If the abundance of herbivorous fish is reduced through fishing, then *d*_*V*_ will be reduced. We also investigated a variant of the model that includes the potential for macroalgae to have direct negative effects on coral recruitment, growth, and/or survival, in addition to competition for space on the reef (see S1 File).

#### Stage-structured macroalgae model (old/large macroalgae are invulnerable to herbivory)

In the second model we add stage-structure to the macroalgae population to include the fact that herbivory is often concentrated on the younger/smaller stages, with the older/larger stages being less vulnerable to herbivory. Thus, in this model *M* is divided into two classes: *M*_*V*_ and *M*_*I*,_ which are the fractions of space occupied by vulnerable and invulnerable macroalgae, respectively. The equations describing the temporal dynamics of this system are:

$$\frac{dC}{dt}=\phi_{C}T+g_{TC}TC-{\gamma g}_{TI}M_{I}C-d_{C}C$$ (3)

$$\frac{dM_{V}}{dt}=\phi_{M}T+r_{M}TM_{I}+g_{TV}TM_{V}-d_{V}M_{V}-\omega M_{V}$$ (4)

$$\frac{dM_{I}}{dt}=\omega M_{V}+g_{TI}TM_{I}+{\gamma g}_{TI}M_{I}C-d_{I}M_{I}$$ (5)

with *T = 1 –C–M*_*V*_*−M*_*I*_. Free space is taken over by vulnerable macroalgae via recruitment of propagules from outside the system (at rate *ϕ*_*M*_), local recruitment by propagules produced by invulnerable macroalgae (at rate *r*_*M*_), and growth and propagule production by vulnerable macroalgae (at combined rate *g*_*TV*_). Loss of macroalgae due to grazing by herbivores is included in *d*_*V*_, the death rate of vulnerable macroalgae (*M*_*V*_). We assume a constant rate of maturation (*ω*) of macroalgae out of the vulnerable class and into the invulnerable class, which implies that the time spent in the vulnerable class is exponentially distributed, with a mean of *1/ω* years (however, with mortality, the average time spent in this class is reduced to *1/(ω +d*_*V*_*)* years). The invulnerable stage of macroalgae (*M*_*I*_) grows laterally over free space at rate *g*_*TI*_, and is lost due to mortality at rate *d*_*I*_ (with *d*_*I*_ *< d*_*V*_). Only the larger, invulnerable stages of macroalgae can grow laterally over coral, at rate *γ g*_*TI*_, where the scaling constant *γ ≤ 1* allows the growth rate of macroalgae over coral to be slower than its growth rate over free space. Coral can take over free space through open recruitment (at rate *ϕ*_*C*_) and through lateral growth at rate *g*_*TC*_, and coral is lost through mortality at rate *d*_*C*_.

#### Model analysis

**Fig 1:**
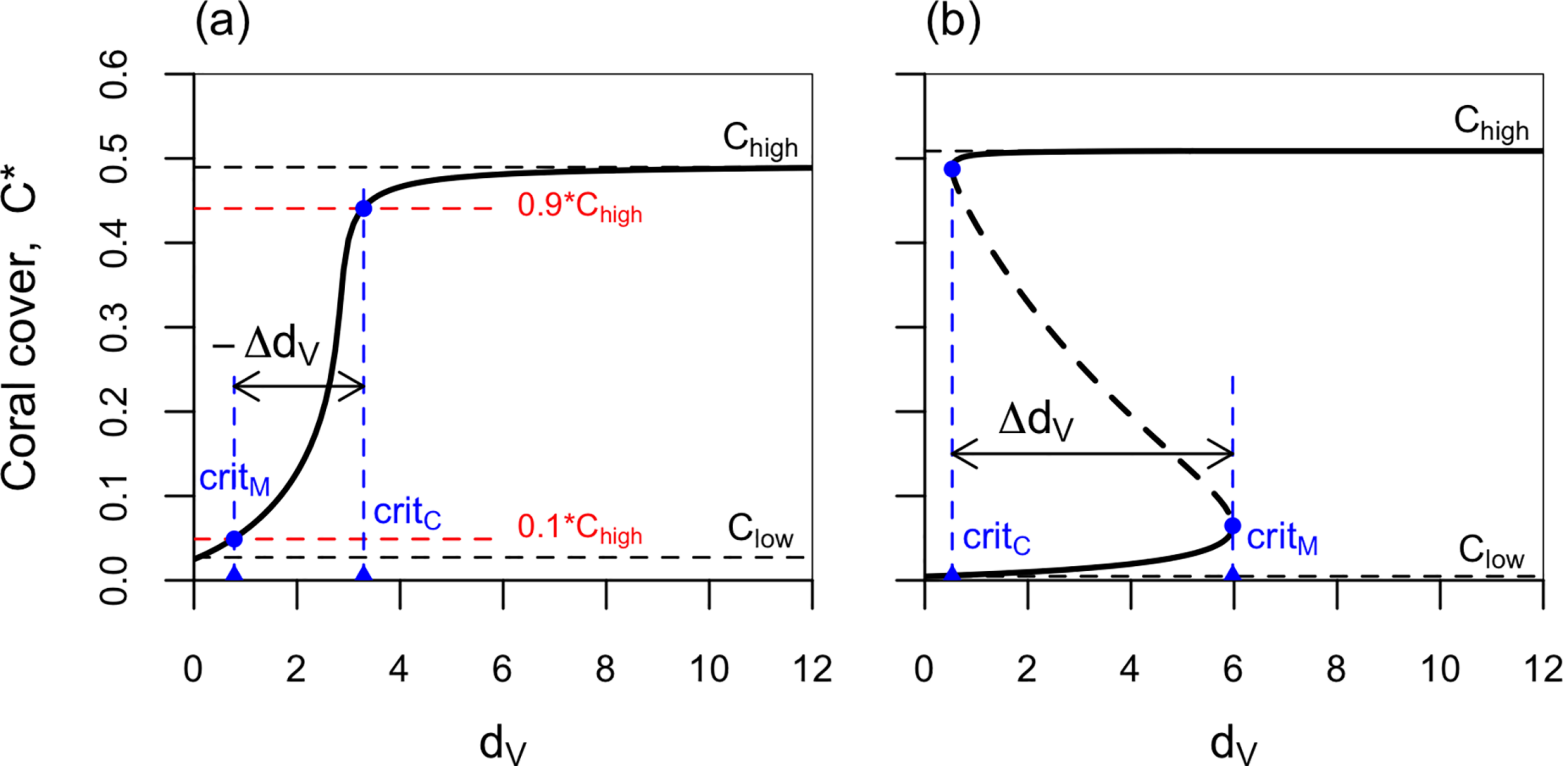
Bifurcation diagrams illustrating model output metrics. In (a), *Δd*_*V*_ *<0* and there is only a single coral steady state for each value of *d*_*V*_. In (b) *Δd*_*V*_ >0 and alternative stable states exist for the values of *d*_*V*_ between *crit*_*C*_ and *crit*_*M*_, and an unstable equilibrium (dashed black line) exists between the two stable equilibria.

We defined feasible ranges of parameters based on the literature, using parameter values derived from empirical studies by Fung et al. [13] and modifying appropriately where our model structure differed (Table 1). These values span the ranges that may be observed in coral reef systems in different parts of the world, allowing us to assess the generality of our results. Bifurcation diagrams, which show the effects of changes in a model parameter on the equilibrium states of the system, were constructed to show the effects of altering *d*_*V*_, the loss rate of macroalgae (e.g., due to herbivory), on the equilibrium fraction of space occupied by coral. We defined four points on the bifurcation diagrams (illustrated in Fig 1A and 1B): *C*_*high*_ is the equilibrium coral cover at very high levels herbivory (i.e., at *d*_*V*_ = 12 y^-1^), *C*_*low*_ is the equilibrium coral cover at very low levels of herbivory (i.e., at *d*_*V*_ = 0.01 y^-1^), *crit*_*C*_ is the lowest value of *d*_*V*_ for which a coral dominated equilibrium state, and *crit*_*M*_ is the highest value of *d*_*V*_ for which a macroalgae-dominated equilibrium state, exist. To be able to define the two critical values, *crit*_*C*_ and *crit*_*M*_, for cases in which there is a smooth transition between the coral-dominated and algae-dominated states, we operationally define crit_C_ for those cases as the lowest value of *d*_*V*_ for which the equilibrium coral cover is *≥ 0*.9*C_*high*_, and *crit*_*M*_ as the highest value *d*_*V*_ for which the equilibrium coral cover is *< 0*.1* C_*high*_. We define *Δd*_*V*_ as (*crit*_*M*_*−crit*_*C*_). If *Δd*_*V*_ > 0, then alternative stable states exist for some range of levels of herbivory, and hysteresis is possible in the system. If *Δd*_*V*_ *< 0*, then coral cover increases monotonically with *d*_*V*_, and there is a single stable equilibrium for all values of *d*_*V*_. The magnitude of *Δd*_*V*_ indicates whether this occurs for a broad or narrow range of parameters. Bifurcation diagrams were constructed using matcont in Matlab.

**Table 1:** Default parameters and parameter ranges.

Parameter	Description	Unstructured model	Stage-structured model
		Default value (range)	Default value (range)
*ϕ*_*C*_	Rate of open recruitment of coral	0.001 (0 to 0.05) y^-1^	0.001 (0 to 0.05) y^-1^
*ϕ*_*M*_	Rate of open recruitment of macroalgae	0.0001 (0 to 0.05) y^-1^	0.0001 (0 to 0.05) y^-1^
*r*_*M*_	Production of vulnerable macroalgae from invulnerable stage	-	0.5 (*g*_*TC*_ to (*g*_*TC*_ + 1)) y^-1^
*g*_*TC*_	Combined rate of growth and local recruitment of corals over free space	0.1 (0 to 0.2) y^-1^	0.1 (0 to 0.2) y^-1^
*g*_*TV*_	Growth of vulnerable macroalgae over free space	-	0.2 (*g*_*TC*_ to (*g*_*TC*_ + 1)) y^-1^
*g*_*TI*_	Growth of invulnerable macroalgae over free space	-	0.4 (*g*_*TC*_ to (*g*_*TC*_ + 1)) y^-1^
*g*_*TM*_	Growth of macroalgae over free space	0.5 (g_TC_ to (*g*_*TC*_ + 1)) y^-1^	-
*γ*	Growth of macroalgae over coral vs. free space.	0.5 (0 to 1)	0.4 (0 to 1)
*d*_*C*_	Death rate of coral	0.05 (0 to 0.1) y^-1^	0.05 (0 to 0.1) y^-1^
*d*_*V*_	Death rate of vulnerable macroalgae	varies (0 to 12)	Varies (0 to 12)
*d*_*I*_	Death rate of invulnerable macroalgae	-	0.4 (*d*_*C*_ to (*d*_*C*_ + 1)) y^-1^
*ω*	Maturation rate of macroalgae from vulnerable to invulnerable class	-	0.5 or 2^a^ (0 to 12) y^-1^

^a^Two “default” values of *ω* were used, to illustrate situations with a relatively long and a relatively short vulnerable macroalgae stage.

For each model we performed both local and global sensitivity analyses to determine the sensitivity of the model output to variation in the parameters. The model output metrics that we investigated were: *hyst* (a binary response variable indicating whether or not alternative stable states and hysteresis are possible for any values of herbivory on macroalgae; i.e., whether or not *Δd*_*V*_ *> 0*), *Δd*_*V*_, *crit*_*C*_, *crit*_*M*_, *C*_*high*_, and *M*_*high*_ (which is defined as the equilibrium cover of macroalgae in the macroalgae-dominated state, i.e., when *d*_*V*_ is very low).

For local (one-at-a-time) sensitivity analysis (LSA), we calculated the change in each output metric for a small change in each parameter (a 10% increase), with all other parameters set to their default values. We performed LSA using the default parameter values in Table 1, and also using 1000 random combinations of “default sets” from the feasible parameter ranges. See S2 File for details of our LSA approach.

We employed two methods for global sensitivity analysis (GSA), which together produce three indices of parameter importance: (i) the Random Forest approach described in Harper et al. [26], and (ii) the variance-based approach of Saltelli et al. [28], which calculates Sobol’s first order and total effect indices. To perform GSA, for each model we constructed a set of 20,000 random parameter combinations, in which the value for each parameter was drawn from a uniform distribution spanning its feasible range of values in Table 1. For the Random Forest approach, a dataset was created in which each of the output metrics (listed above) was calculated for each parameter combination, and classification and regression trees (CART) [29,30] were used to recursively split the dataset into two parts, based on the value of a parameter that maximized the homogeneity of the two subsets. Random Forests create multiple trees from subsets of the dataset and subsets of the parameters, in order to quantify both the classification error and the importance of the parameters [26,30]. This approach to GSA includes the effects of any non-linear interactions among the parameters in its estimate of parameter importance. The Sobol/Saltelli method for GSA is a variance-based method [27,28] that determines the relative contribution of each parameter to the variation in each output metric. Sobol’s first order index does not include interactions between the parameters, while Sobol’s total effect index includes the effects of the interactions of each parameter with all other parameters [25]. See S3 File for details of our approaches for GSA. For both LSA and GSA, we excluded parameter combinations for which coral or algae always dominate the space on the reef regardless of the level of herbivory, as described in S3 File.

## Results

### Macroalgae stage-structure promotes alternative stable states and hysteresis

**Fig 2:**
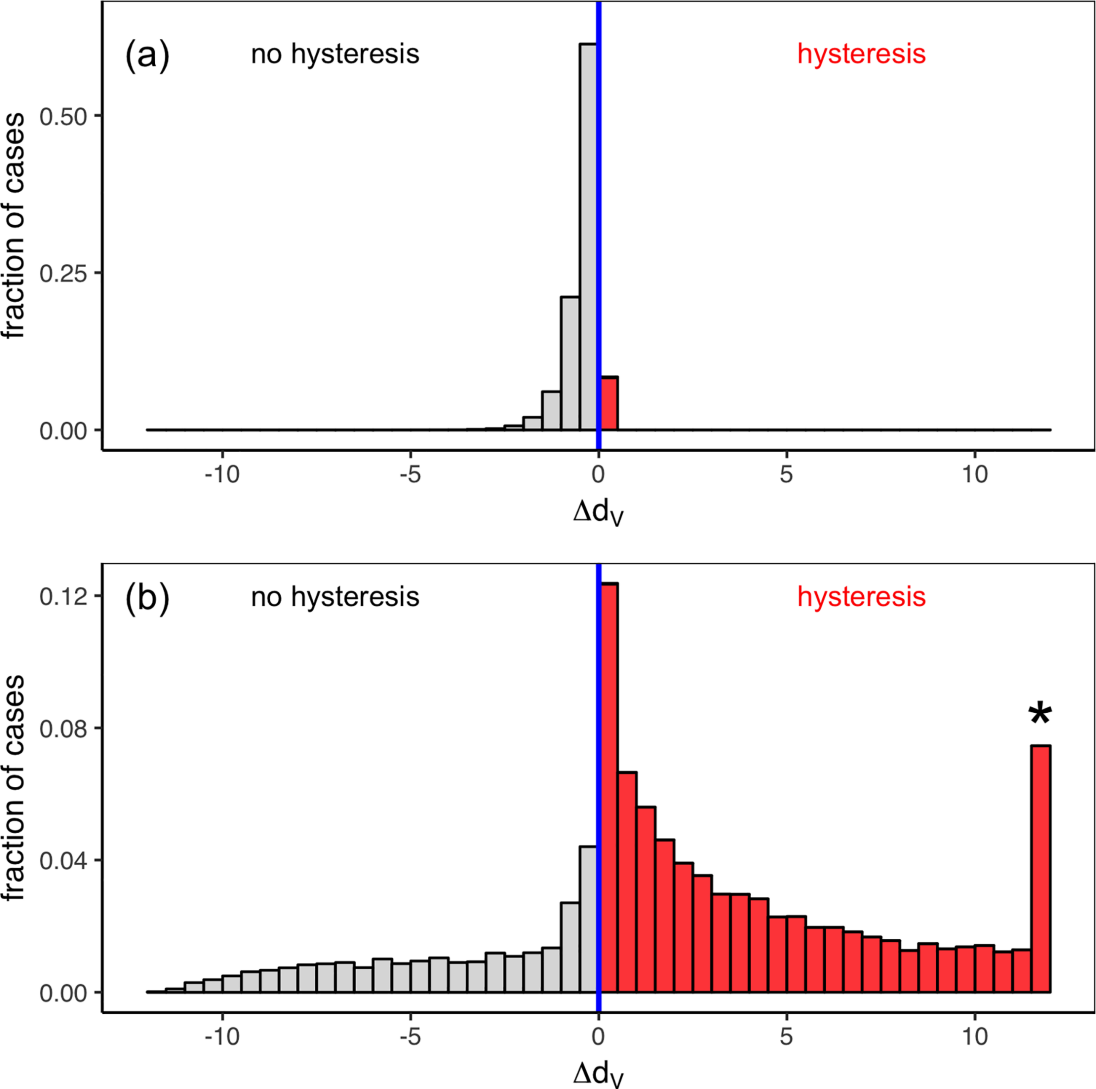
**Histograms showing the distribution of values of *Δd***_***V***_, for 20,000 random combinations of parameters for (a) the unstructured macroalgae model, and (b) the stage-structured macroalgae model (the * includes all parameter combinations for which *Δd*_*V*_ ≥ 12).

**Fig 3:**
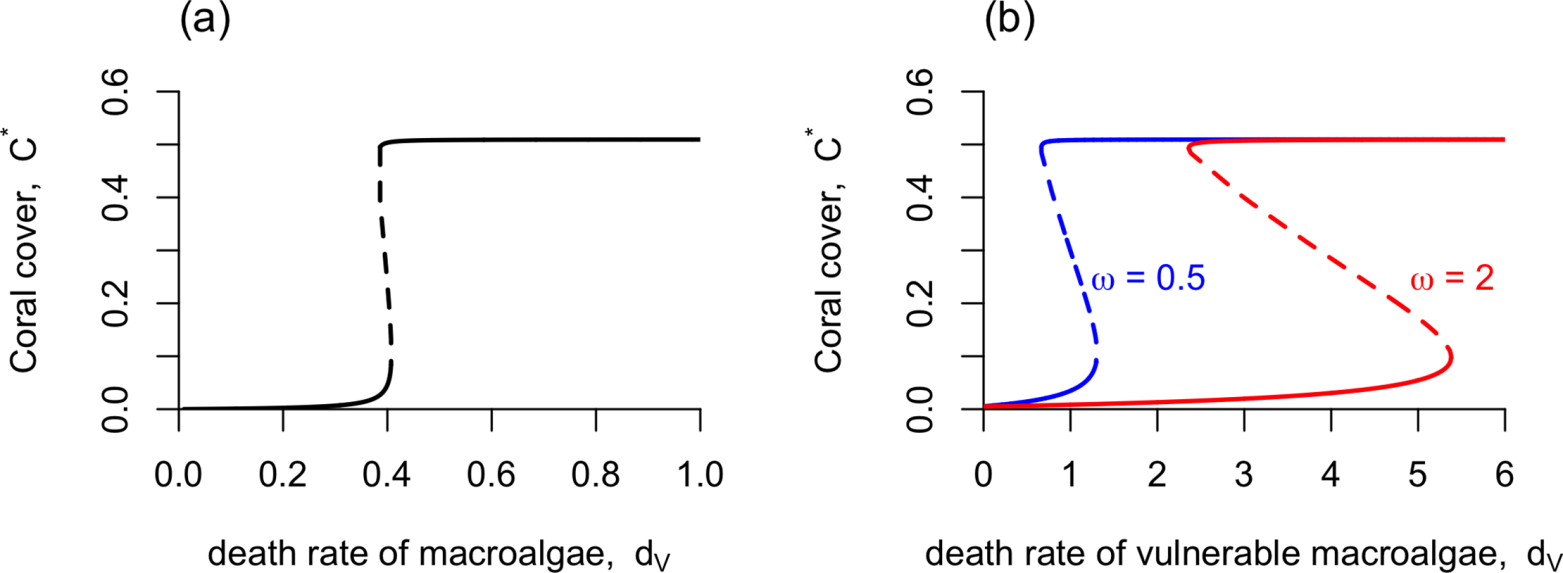
**Bifurcation diagrams** for (a) the unstructured macroalgae model, and (b) the stage-structured macroalgae model, showing the steady-state coral cover (C^*^) as a function of the death rate of vulnerable macroalgae (*d*_*V*_). Solid lines represent stable equilibria, and dashed lines represent unstable equilibria. The default parameters in Table 1 were used.

Alternative stable states and hysteresis are very unlikely outcomes for the Unstructured Macroalgae Model (Figs 2A and 3A), occurring at some level of herbivory in only about 6% of the randomly selected sets of parameters, and for only a very narrow range of values of herbivory (Fig 2A; the maximum *Δd*_*V*_ observed was only 0.37 y^-1^). Despite the unlikelihood of alternative stable states, this model readily produces abrupt state transitions following small changes in the level of herbivory. The transition from a coral-dominated state to an algae-dominated state occurs over a very narrow range of herbivory for most parameter combinations (i.e., *|Δd*_*V*_*|* < 0.5 y^-1^ in ~70% of the parameter combinations that we examined; Fig 2A). In contrast, alternative stable states and hysteresis are common outcomes in the Stage-Structured Macroalgae Model, occurring in ~75% of the parameter combinations tested, spanning wide ranges of levels of herbivory (Figs 2B and 3B).

### Effects of parameters on model outcomes

We concentrate here on the results of the Stage-Structured Macroalgae Model; results for the Unstructured Macroalgae Model are provided in Appendices 2 and 3.

#### Local sensitivity analysis (LSA)

**Fig 4:**
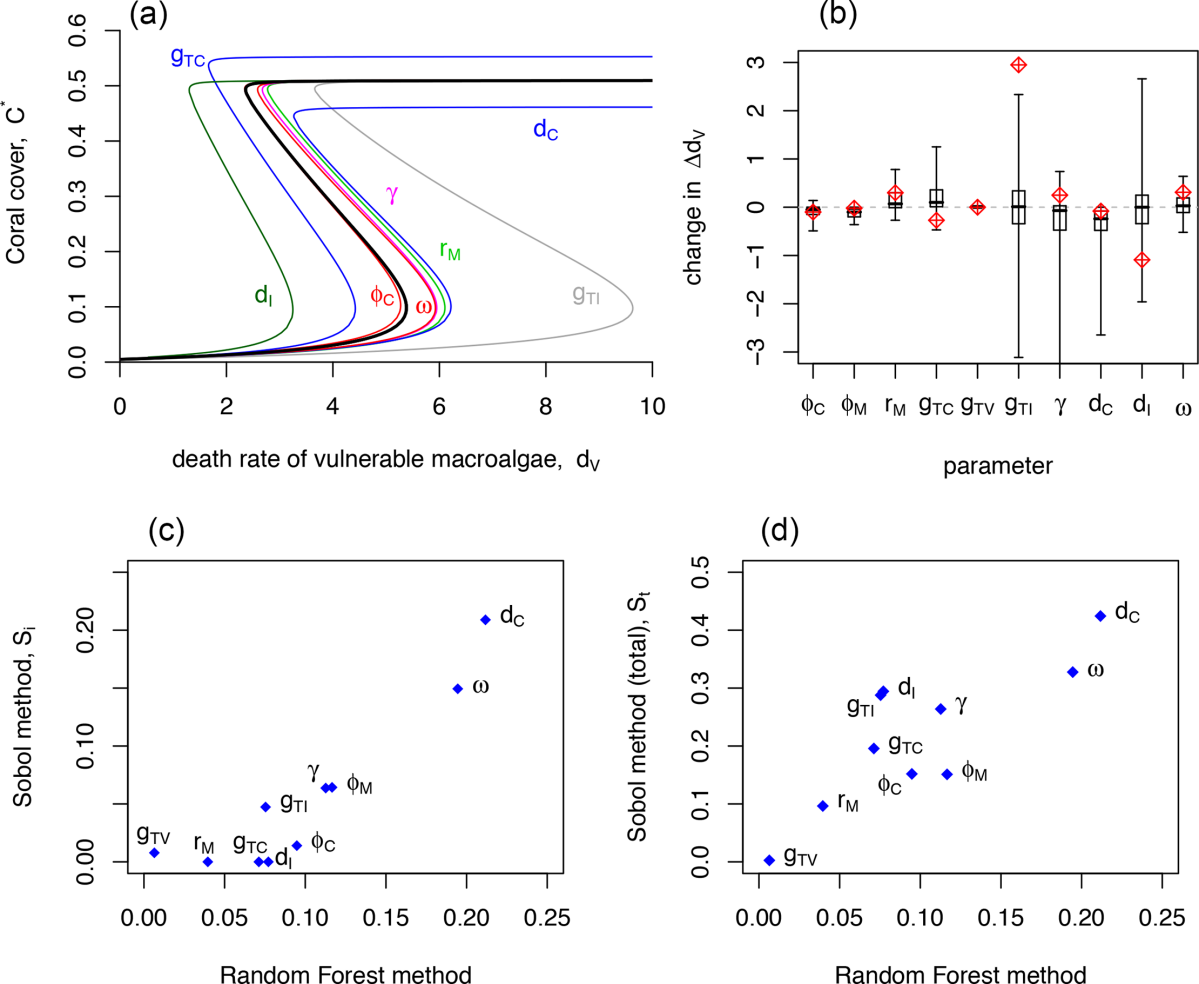
Sensitivity analysis. (a) and (b) Results of local sensitivity analyses on *Δd*_*V*_ for the Stage-structured Macroalgae Model. (a) Bifurcation diagrams showing the effects of a one-at-a-time 10% increase in the parameters from the default set (with *ω* = 2, black line; *ϕ*_M_ and *g*_*TV*_ lines are indistinguishable from the default set). (b) Change in *Δd*_*V*_ resulting from a one-at-a-time 10% increase in each parameter. The stem-and-whisker plots show the median (horizontal black lines), the 25^th^ and 75^th^ quantiles (boxes), and the 5^th^ and 95^th^ quantiles (lower and upper bars) of the change in *Δd*_*V*_ resulting from a 10% one-at-a-time increase using 1000 randomly sampled “default sets”. The red diamonds indicate the default set listed in Table 1. (c) and (d) Comparison of two methods for Global Sensitivity Analysis showing the importance of the parameters in the Stage-structured Macroalgae Model in determining *Δd*_*V*_: (c) comparing the Random Forest method and the first-order sensitivity index (i.e., Sobol’s Index; *S*_*i*_), and (d) comparing the Random Forest method and the total importance metrics from Sobol’s method, *S*_*t*,*i*_.

Fig 4A shows the effects of a one-at-a-time 10% increase in each parameter from the default values in Table 1, suggesting that the growth and death rates of invulnerable algae (*g*_*TI*_ and *d*_*I*_) might be the most influential parameters determining the presence and magnitude of hysteresis (*Δd*_*V*_) (see also Fig 4A in S2 File). This result, however, depends heavily on the choice of the default set of parameters as the effect of small changes in some of the parameters depends on the values of the others in the model (Fig 4B). For example, for the default parameters, a 10% increase in *g*_*TI*_ shifts both *crit*_*C*_ and *crit*_*M*_ to higher values of *d*_*V*_, leading to a dramatic increase in *Δd*_*V*_ (Fig 4A). Fig 4B reveals, however, that our default parameter set is an outlier in that regard, in that a small increase in *g*_*TI*_ leads to an unusually large increase in *Δd*_*V*_. A small increase in this parameter can have widely different effects on *Δd*_*V*_ depending on the values of the other parameters (large error bars in Fig 4B); in some cases increasing the magnitude of hysteresis, and in other cases shifting the system from one in which alternative stable states occur for wide ranges of herbivory to one in which the coral cover increases vary gradually with increasing herbivory (i.e., a shift from positive to negative *Δd*_*V*_). Small changes in the death rate of invulnerable macroalgae (*d*_*I*_) have similarly highly variable effects on *Δd*_*V*_ (Fig 4B). Other parameters have more consistent effects, for example, increasing the death rate of coral always reduces the likelihood of alternative stable states because it shifts the system away from a coral-dominated state. Similarly, increasing the external inputs of either corals or macroalgae (*ϕ*_*C*_ or *ϕ*_*M*_) tends to reduce the likelihood of alternative stable states, although this effect is relatively small for the range of parameters investigated. The LSA results are explored further in S2 File.

#### Global sensitivity analysis (GSA)

All three GSA indices of parameter importance agree that the death rate of coral (*d*_*C*_) and the rate of maturation of algae out of the vulnerable stage (*ω*) are the two parameters with the greatest influence on the presence and magnitude of hysteresis (*Δd*_*V*_). Interestingly, these two parameters were not identified as particularly important for the local sensitivity. All of the methods suggest that the two parameters governing the rate of local production of vulnerable macroalgae (*g*_*TV*_ and *r*_*M*_), have relatively little influence on *Δd*_*V*_. The remaining parameters all contributed to *Δd*_*V*_ to some degree (Fig 4C and 4D). The Random Forest analysis explained approximately 75% of the variance in *Δd*_*V*_, and the best-fit pruned tree produced by CART (Fig A10 in S3 File) indicates that *Δd*_*V*_ depends on interactions between several of the parameters. Specifically, alternative stable states tend to occur (i.e., positive *Δd*_*V*_) when the coral death rate is low and the maturation rate of algae out of the vulnerable stage is high. Alternatively, the situation in which coral cover increases gradually with increasing herbivory (i.e., negative *Δd*_*V*_) is most likely to occur when the death rate of coral is relatively high and either invulnerable macroalgae readily grow over coral (high *γ*), or in the case of low *γ*, when the death rate of invulnerable macroalgae is also relatively low. These are conditions that reduce the dominance of either of the taxa.

The parameter importance rankings for the two variance-based global sensitivity indices, Sobol’s first order (*S*_*i*_) and total effect index (*S*_*t*,*i*_), agree to a large degree with those of the Random Forest analysis, but there are some notable discrepancies (Fig 4C and 4D). The rankings of parameter importance for the Random Forest method are more in agreement with those of Sobol’s total effect index (*S*_*t*,*i*_: Fig 4D) than with Sobol’s first order effect index (*S*_*i*_: Fig 4C). This is as expected because both the Random Forest method and *S*_*t*,*i*_ include the effects of interactions between the parameters on the output metric, while *S*_*i*_ does not. The three parameters for which there are discrepancies in the importance rankings between *S*_*t*,*i*_ and the Random Forest method are *g*_*TI*_, *γ*, and *d*_*I*_, with *S*_*t*,*i*_ giving higher importance rankings than the Random Forest method. This is understandable, because these are the three parameters for which the local sensitivity analysis found highly variable changes in *Δd*_*V*_ in response to small changes in the parameter (Fig 4B; see also Fig A5c in S2 File).

## Discussion

Following large disturbances some coral reefs have switched rapidly from a coral-dominated state to a state dominated by fleshy macroalgae [31,32]. Such phase shifts can persist for decades and it has been hypothesized that they could represent shifts between alternate stable states or alternate basins of attraction [11,18,32]. Many of the mechanisms that could lead to alternate stable states have not been explored theoretically, which is surprising given the level of debate this topic has generated [8,9,33]. We found that including a well-known biological detail in a simple model of coral-algae dynamics greatly enhanced the likelihood of alternative stable states. Specifically, when algae can outgrow the risk of herbivory, our model suggests that a pulse perturbation (e.g., a rapid decrease in coral cover), or a small change in the level of herbivory, could cause a reef to move rapidly from a coral-dominated equilibrium to an algae-dominated equilibrium. The qualitative results of our study are not entirely surprising. Many organisms reach a life stage where they become relatively invulnerable to predation and this is a key mechanism leading to alternate attractors in a variety of systems [3,34,35]. However, the magnitude of the region of hysteresis we observed was striking. Further, our sensitivity analyses indicate that stage-structure in the susceptibility of algae to herbivores can lead to bistability across a very wide range of parameter space, suggesting that bistability in coral-algae phase shifts may not be confined to highly degraded reefs in a single geographic region as has been hypothesized based on the results of previous models [8,10].

The two methods for GSA agree that the two parameters in the stage-structured macroalgae model that have the greatest influence on the range of levels of herbivory over which alternative stables states occur (i.e., on *Δd*_*V*_) are the death rate of coral, and the rate of maturation of macroalgae out of the vulnerable class, with bistability most likely to occur with long-lived coral (low *d*_*C*_) and a vulnerable macroalgae stage that is short in duration (high *ω*). These are conditions in which the system is most likely to stay in either a coral-dominated, or an algae-dominated, state once it gets there. Overall, we found that the rankings of parameter importance from the Random Forest method agreed quite closely with Sobol’s total sensitivity index, *S*_*t*,*i*_, as both of these metrics include both the direct effects of the parameters on the output metrics, and also the effects of interactions among the parameters. The parameters for which there were discrepancies between the rankings of these two models (with Sobol’s total sensitivity index giving higher rankings than the Random Forest method) were also the parameters for which the local sensitivity analysis found high levels of variability. These discrepancies can be explained by the fact that *S*_*t*,*i*_ is based only on variability in the output metrics, whereas the Random Forest method considers the ability to predict the response variable based on the parameter values.

To the best of our knowledge, this is the first comparison of the Random Forest approach to GSA [26] and the variance-based Sobol’s method [28]. The strong agreement between these two approaches may lead to the Random Forest method being more commonly adopted, for both ecological models and in the broader scientific literature, because the Random Forest method is very straight-forward to implement, and is less computationally intense (i.e., it requires fewer runs of the model or numerical computations of the equilibrium values to calculate the output metrics). To calculate the global sensitivity using *n* parameter combinations, the Random Forest approach requires *n* runs of the model, while the “efficient” implementation of Sobol’s method [28] requires (2+k)*n runs of the model, where *k* is the number of parameters.

A key assumption in our stage-structured model is that macroalgae reach a stage where they are completely invulnerable to herbivores. This assumption is based on the observation that most herbivorous fishes on coral reefs feed on the small, vulnerable stages of macroalgae, and algal turf (multi-species assemblages of filamentous algae, macroalgal spores, microalgae, and detritus), while avoiding large seaweeds [36]. Indeed, many common seaweeds on algae-dominated reefs are unpalatable due to chemical and physical defenses [37] and are therefore more likely to be removed as propagules and young juveniles living within algal turf than as adults. Nonetheless, complete invulnerability is an oversimplification as many species of macroalgae are fed on by a small group of specialized herbivorous fishes [20,38] and, in some systems, by certain species of echinoids [39,40]. Importantly, macroalgae browsing fishes are often functionally absent from heavily fished systems [41]. Thus, our stage-structured model may be most applicable to systems where heavy fishing has resulted in the functional elimination of macroalgae browsers. Future models that include both turf grazers and macroalgae browsers will be especially useful for understanding how diversity within the herbivore guild influences the dynamics of coral reef ecosystems and how human activities can alter these dynamics.

In contrast to other models of coral-algae phase shifts, reefs in our stage-structured model become locked in a macroalgae-dominated state—not because of inherently low herbivory due to the dilution of grazing intensity—but because herbivores are unable to utilize mature macroalgae as a food resource. Differentiating between these two potential mechanisms as drivers of alternate states on coral reefs is important because they suggest very different management strategies for reversing phase shifts to macroalgae dominance. For example, manually removing macroalgae, a common management strategy on some coral reefs, is likely to be most successful if the primary mechanism maintaining these reefs in an algae-dominated state is the resistance of adult macroalgae to herbivory. However, if dilution of grazing intensity is an important mechanism stabilizing the algae-dominated state, then combining algae removals with outplants of living coral colonies would likely enhance restoration efforts by helping herbivores prevent the re-establishment of algae by concentrating their grazing impacts into smaller areas. In addition to identifying important feedbacks that can maintain a system in an alternate state, ecological models can help guide management by identifying parameters that are particularly important drivers of system dynamics. The most important parameters in our model include the transition rate from vulnerable to invulnerable algae and coral mortality, with low transition rates and low coral mortality favoring a coral-dominated state. This suggests that reducing chronic stressors to corals and nutrient pollution that enhance the growth rates of algae are important management goals that can help reduce the likelihood that a reef will undergo a shift to an alternate stable state.

## Supporting information

S1 File Variant of model with direct negative effects by macroalgae on coral. (PDF)

S2 File Details of local sensitivity analysis. (PDF)

S3 File Details of global sensitivity analysis. (PDF)
