# Supplementary material for: Macroalgae size refuge from herbivory promotes alternative stable states on coral reefs
Source: PLoS One. 2018 Sep 18;13(9):e0202273. doi: 10.1371/journal.pone.0202273 (PMC6143192; doi:10.1371/journal.pone.0202273)
Supplement: S1 File — (PDF) [file pone.0202273.s001.pdf]

S1 File. Variant of model with direct negative effects by macroalgae on coral

Here we present a variant of the model that includes the potential for macroalgae to exert direct negative effects on coral (similar to [1]), in addition to competition for space on the reef. The parameters ψ_R, ψ_G, and ψ_D allow us to include the potential for macroalgae to have direct negative effects on coral recruitment, growth, and/or survival, respectively. These effects can include allelopathy, whiplash, shading, etc. We assume that the magnitudes of these effects scale linearly with the fraction of space occupied by macroalgae, (with slopes ψ_R, ψ_G, and ψ_D for coral recruitment, growth, and death, respectively).

Note: other models assume that macroalgae propagules are a component of turf algae, and therefore macroalgae grows from turf. Our model can also represent this scenario if ϕ_M is interpreted as comprising the rates of both open recruitment of macroalgae to turf and the growth of macroalgae from turf.

Description of models

Unstructured model

The equations for the unstructured model with direct negative effects of macroalgae on coral are:

$$dC/dt = \phi_C (1 - \psi_R M) T + g_{TC} (1 - \psi_G M) T C - \gamma g_{TM} M C - d_C (1 + \psi_D M) C$$

recruitment growth overgrowth death

$$dM/dt = \phi_M T + g_{TM} T M + \gamma g_{TM} M C - d_V M$$

recruitment growth overgrowth loss/herbivory

with $T = 1 - C - M$

Stage-structured model

For the Stage-structured model, we assume that only the invulnerable class of macroalgae has the potential to have other direct negative effects on coral recruitment, growth, and/or death (at rates ψ_R, ψ_G, and/or ψ_D, respectively).

The equations for the stage-structured model with direct negative effects of the invulnerable macroalgae class on coral are:

$dC/dt = \phi_C (1 - \psi_R M_I) T + g_{TC} (1 - \psi_G M_I) T C - \gamma g_{TI} M_I C - d_C (1 + \psi_D M_I) C$

recruitment growth overgrowth death

$dM_V/dt = \phi_M T + r_M T M_I + g_{TV} T M_V - d_V M_V - \omega M_V$

recruitment reproduction growth loss/grazing maturation

$dM_I/dt = \omega M_V + g_{TI} T M_I + \gamma g_{TI} M_I C - d_I M_I$

maturation growth overgrowth loss

with $T = 1 - C - M_V - M_I$

Results

Unstructured model

Fig A1 shows that for the unstructured model, the direct negative effects of macroalgae on coral have little effect. Increasing ψ_R, ψ_G, and/or ψ_D only slightly increases the region of parameter space for which alternative stable state and hysteresis can occur.

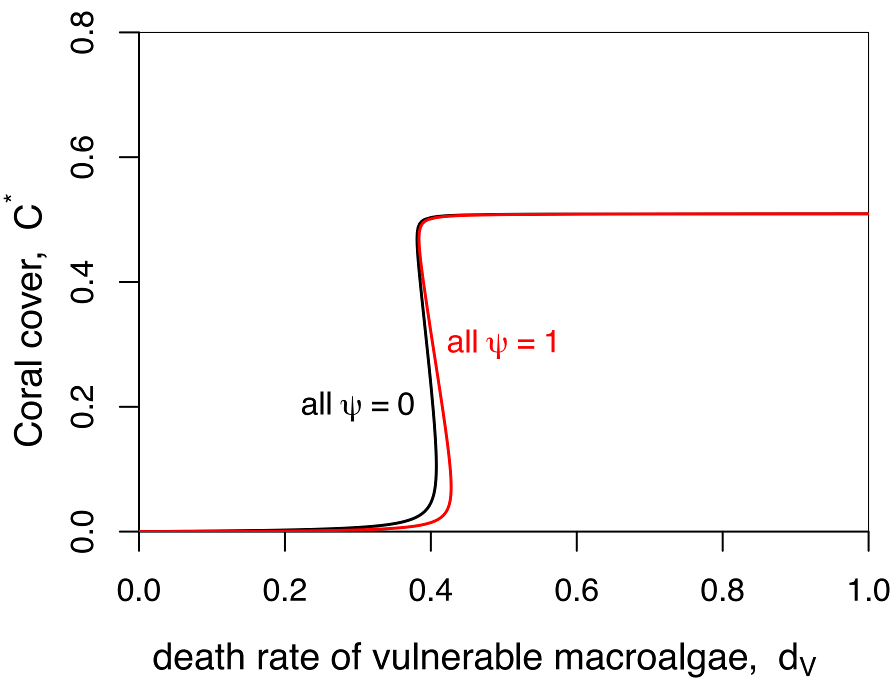

Fig A1. Bifurcation diagrams for unstructured model with $\psi_R = \psi_G = \psi_D = 0$ and $\psi_R = \psi_G = \psi_D = 1$. All other parameters are set to the default values in Table 1 in the main text.

Stage-structured macroalgae model

Fig A2 shows that the direct negative effects of macroalgae on coral can increase the likelihood of alternative stable state and hysteresis in the stage-structured model, especially if these effects are simultaneously included in the coral recruitment, growth, and death rates simultaneously (ψ_R, ψ_G, and ψ_D all > 0).

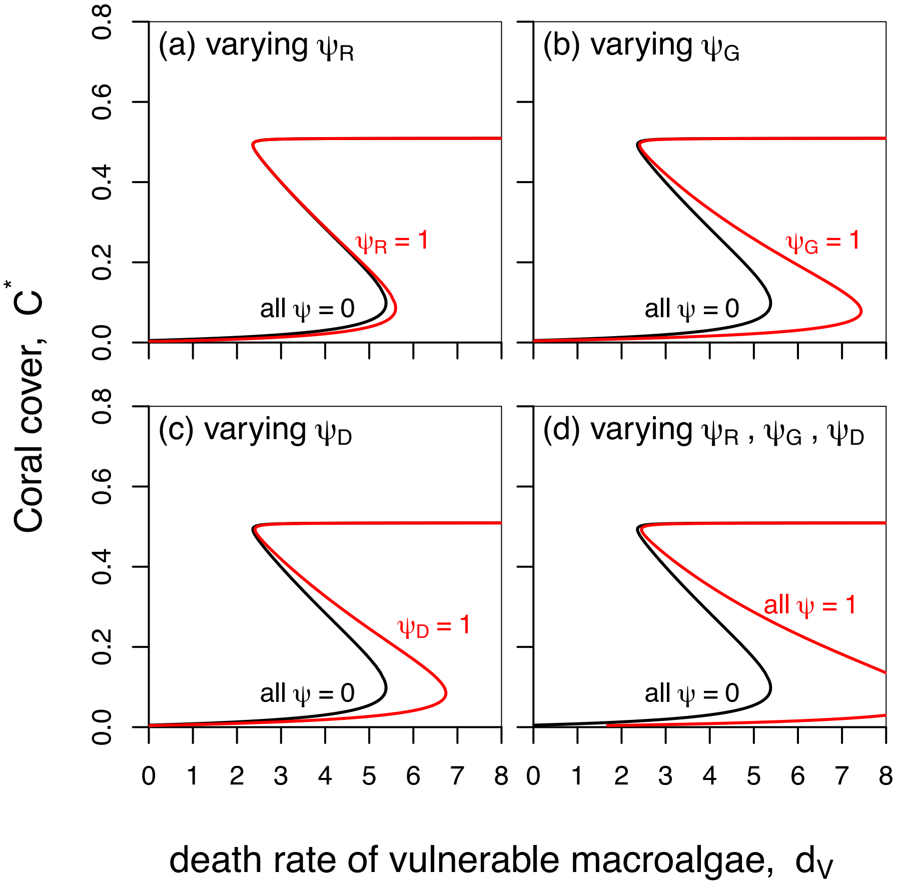

Fig A2. Bifurcation diagrams for stage-structured macroalgae model, showing the effects of increasing values of ψ_R, ψ_G, or ψ_D individually, or simultaneously. All other parameters are set to the default values in Table 1 in the main text.

References

1. Fung T, Seymour RM, Johnson CR. Alternative stable states and phase shifts in coral reefs under anthropogenic stress. Ecology. 2011;92: 967–82. Available: http://www.ncbi.nlm.nih.gov/pubmed/21661558
