# Supplementary material for: Macroalgae size refuge from herbivory promotes alternative stable states on coral reefs
Source: PLoS One. 2018 Sep 18;13(9):e0202273. doi: 10.1371/journal.pone.0202273 (PMC6143192; doi:10.1371/journal.pone.0202273)
Supplement: S2 File — (PDF) [file pone.0202273.s002.pdf]

S2 File. Details of local sensitivity analysis

Effects of changing individual parameters in the model

The bifurcation diagrams in Fig A3 (for the Unstructured Model) and Fig A4 (for the Stage-structured Macroalgae Model) show the effects of increasing or decreasing individual parameters one at a time, by an amount larger than the 10% shown in Fig 4a of the main text.

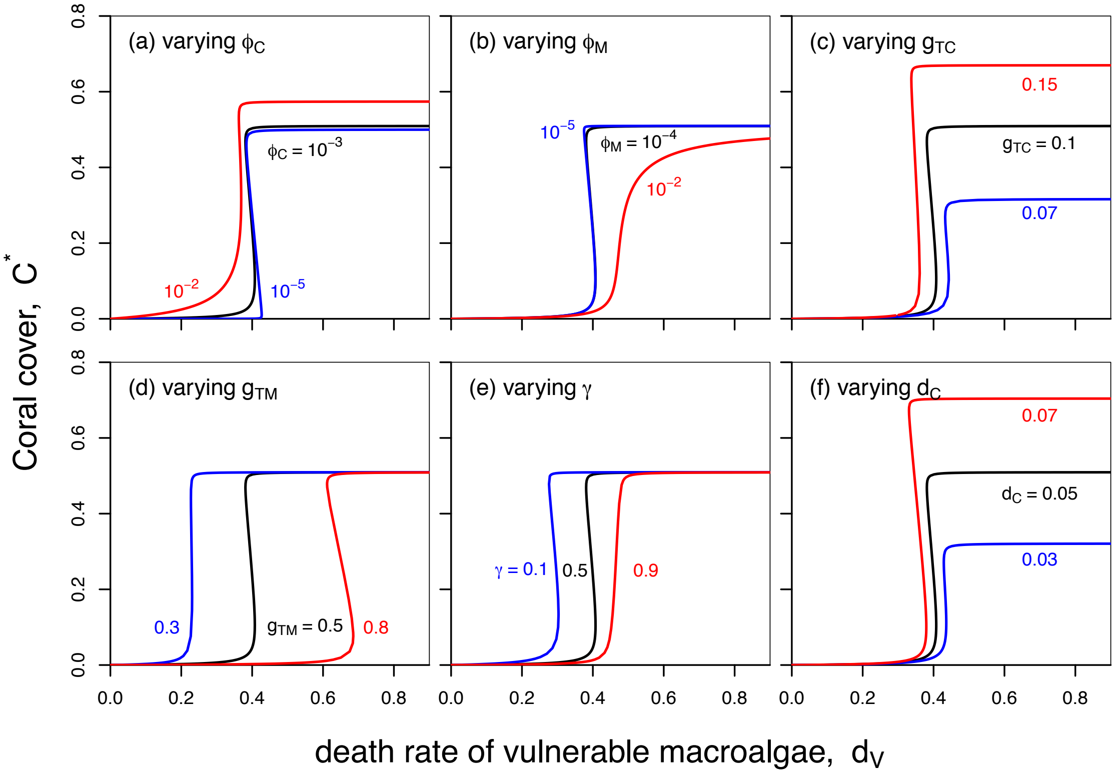

Fig A3. **Bifurcation diagrams for Unstructured Model**, showing the effects of increasing (red lines) or decreasing (blue lines) each parameter individually from the baseline model (black line) on the equilibrium fraction coral cover. The amounts by which the parameters were changed were chosen for illustrative purposes.

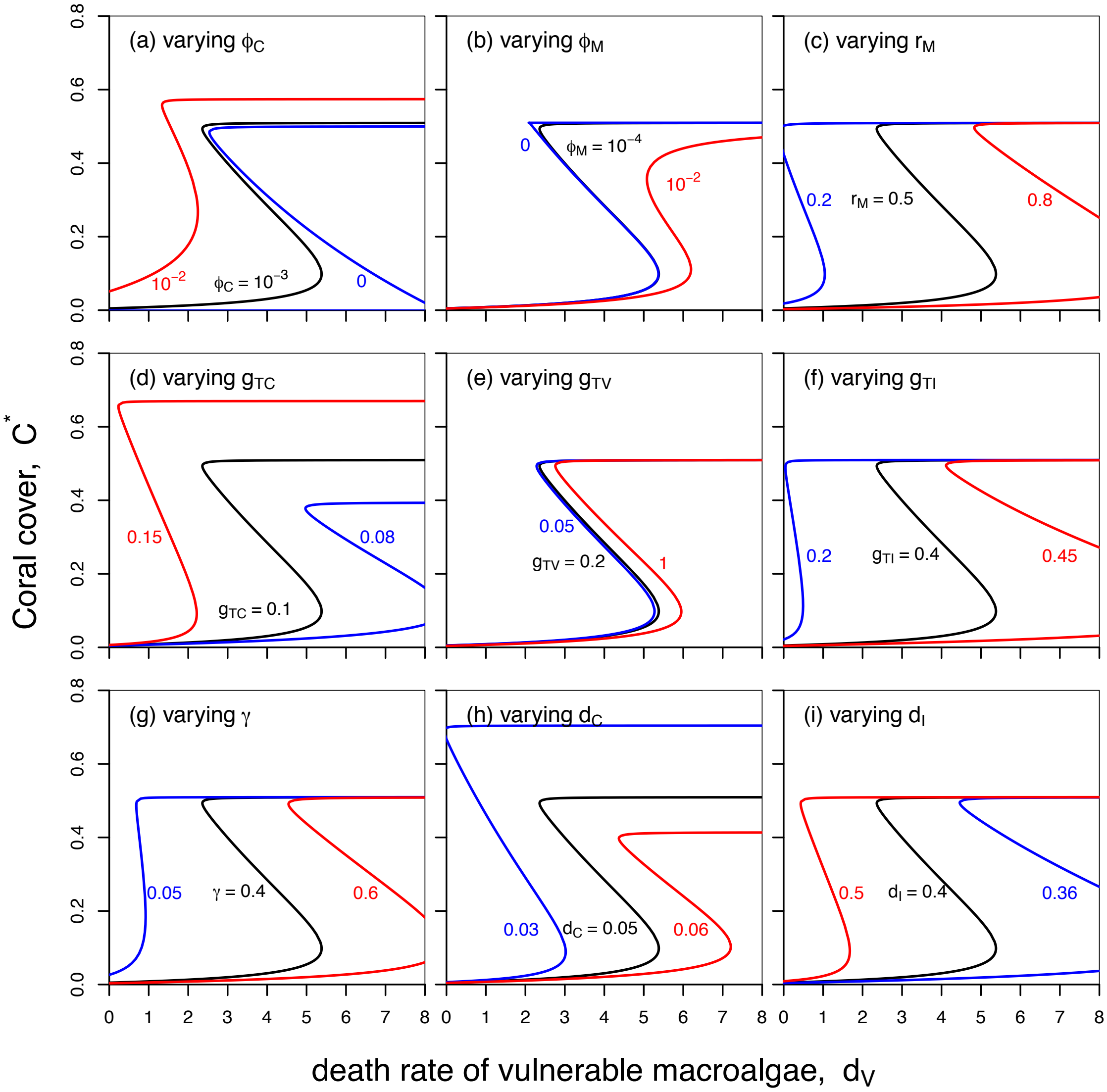

Fig A4. Bifurcation diagrams for the stage-structured macroalgae model, showing the effects of increasing (red lines) or decreasing (blue lines) each parameter individually from the baseline model (black line) on the equilibrium fraction coral cover. The amounts by which the parameters were changed were chosen for illustrative purposes (rather than the 10% one-at-a-time increase shown in Fig 4a in the main text).

Details of Local Sensitivity Analysis

The method that we used to generate Fig 4b in the main text is a variant of the Morris one-at-a-time (OAT) method for local sensitivity analysis [1,2]. We modified this method in two ways. First, the Morris method divides each parameter axis into *p-1* equally-spaced intervals (e.g. if $p = 5$, then for the parameter γ in our model this method the potential values used would be {0, 0.25, 0.5, 0.75, 1}). The Morris method randomly selects from these sets of potential values for each parameter, and then performs a one-at-a-time perturbation by adding a small perturbation to a parameter i in random order. Instead of using this grid-based approach, we employed the Monte-Carlo sampling method commonly used in global sensitivity analysis to select random combinations of parameters from the realistic range. That is, in our version, γ can take on any value in its feasible range from 0 to 1. Theoretically, this should not affect our results. We then perturb the values of all parameters, one at a time, from the randomly-selected default set by adding 10% to each parameter in turn. As described in the main text, for the local sensitivity analysis, we randomly sampled 1000 combinations of parameters (using a uniform distribution) from the feasible ranges of parameter values given in Table 1 of the main text.

The second modification that we made to the Morris method is in the detail of what we calculated for our metric of sensitivity. Morris's method calculates the elementary effect (EE_i) for each parameter i for each combination in its "default sets" [2]:

$$EE_i = \frac{1}{\tau_y} \frac{f(x_1^*, \cdots, x_i^*(1+\delta), \cdots, x_k^*) - f(x_1^*, \cdots, x_i^*, \cdots, x_k^*)}{x_i^* \delta}$$

where $\{x_i^*\}$ is the randomly-selected "default set" of parameters, and $f(\{x_i^*\})$ is the output metric calculated at those parameter values. τ_y is defined as "the change in the output that one would consider to be significant or representative" [p. 85 in 2]. This is relevant when comparing

sensitivities between different output metrics, which we do not discuss here, so we set τ_y to 1. The modification that we made to this measure of sensitivity is that in Fig 4b in the main text we used only the change in the output metric (i.e. the numerator of EE_i, for $\delta = 0.1$ for each parameter i) as our measure of S_i^{local}, because this is what is most readily observed in the bifurcation diagrams of Fig 4a of the main text. In Fig A6 we also show the results in terms of EE_i for $\delta = 0.1$, which is an estimate of the slope of Δd_V with respect to parameter i.

As described in Wainwright et al. [2] we calculated the mean, the mean of the absolute values, and the standard deviation of both EE_i (Fig A6) and our measure of S_i^{local} (i.e. the numerator of EE_i; Fig. A5). Saltelli et al. [3] suggests that low values for the mean of the absolute value of these measures of local sensitivity indicate non-influential parameters, and that high values of the standard deviation can indicate interactions between the parameters or nonlinear effects of the parameters on the output metric. The analysis for S_i^{local} in Fig A5 suggests that 10% increases in the parameters g_{TI}, γ, and d_I have the 3 largest effects on the magnitude of the change in Δd_V, and also have the 3 most variable impacts on Δd_V. In contrast, the analysis for EE_i in Fig A6 suggests that ϕ_C, ϕ_M, and d_C have the 3 greatest effects on the slope of the change in Δd_V in response to a small change in these parameters, and the 3 most variable impacts on this slope. ϕ_M and ϕ_C are the parameters with the two smallest values of all of the parameters, so their large effect as measured by this metric may be due in part to division by a very small denominator (Fig A4 shows the effects of changes in these parameters over several orders of magnitude). Fig A4 reveals that very small changes in d_C do in fact lead to large changes in Δd_V. Neither of these metrics identifies ω as a particularly influential parameter, despite the fact that the Global Sensitivity Analyses suggests that it is one of the two most influential parameters determining Δd_V.

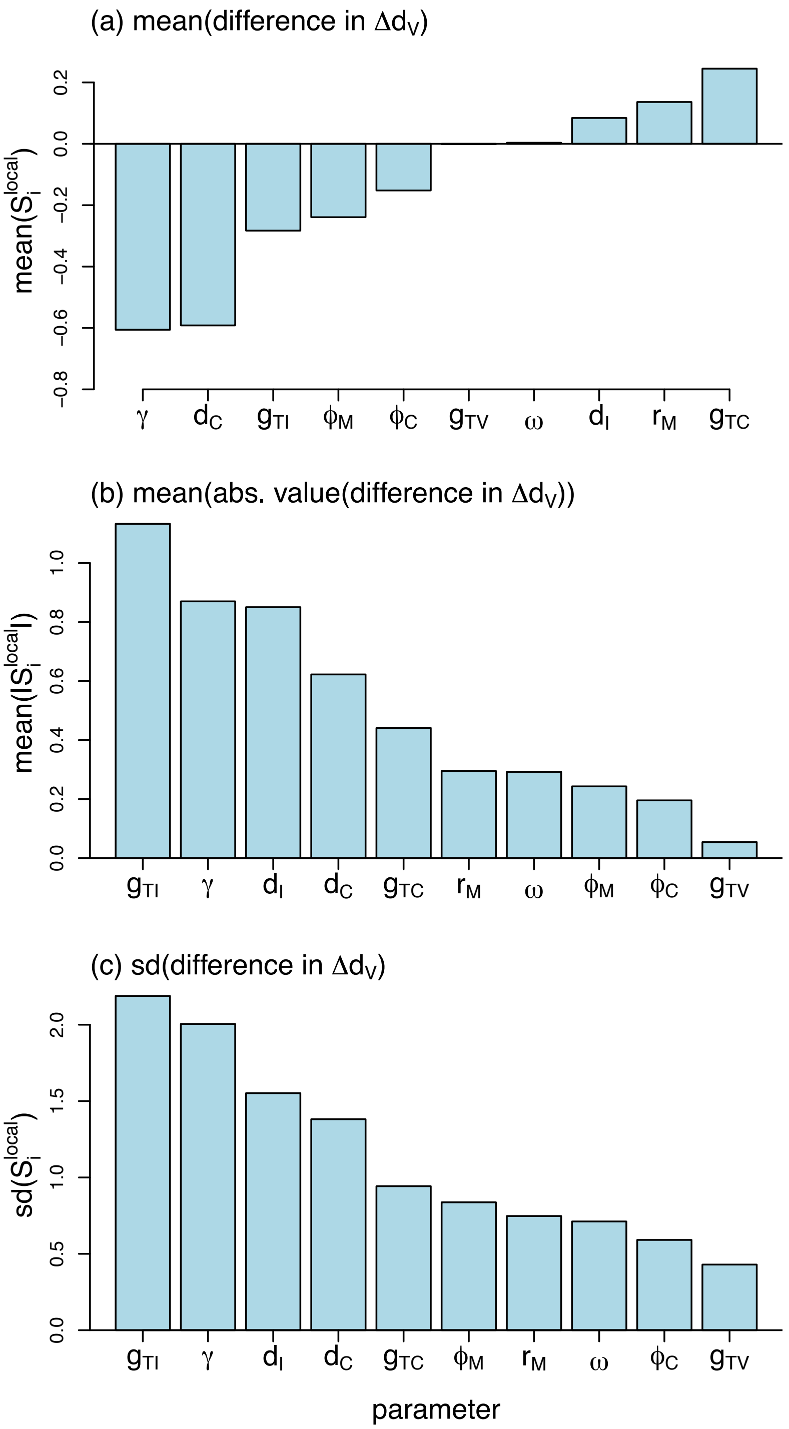

Fig A5. Local sensitivity analysis for the stage-structured macroalgae model, using S_i^{local}, in response to a 10% one-at-a-time increase in each parameter as the measure of local sensitivity,

(i.e. the numerator of EE_i as in Fig 4b of the main text). Shown are (a) the mean, (b) the mean of the absolute values, and (c) the standard deviation of S_i^{local} for a sample of 1000 parameter sets drawn from the feasible ranges of parameters, subject to the constraints described in the text.

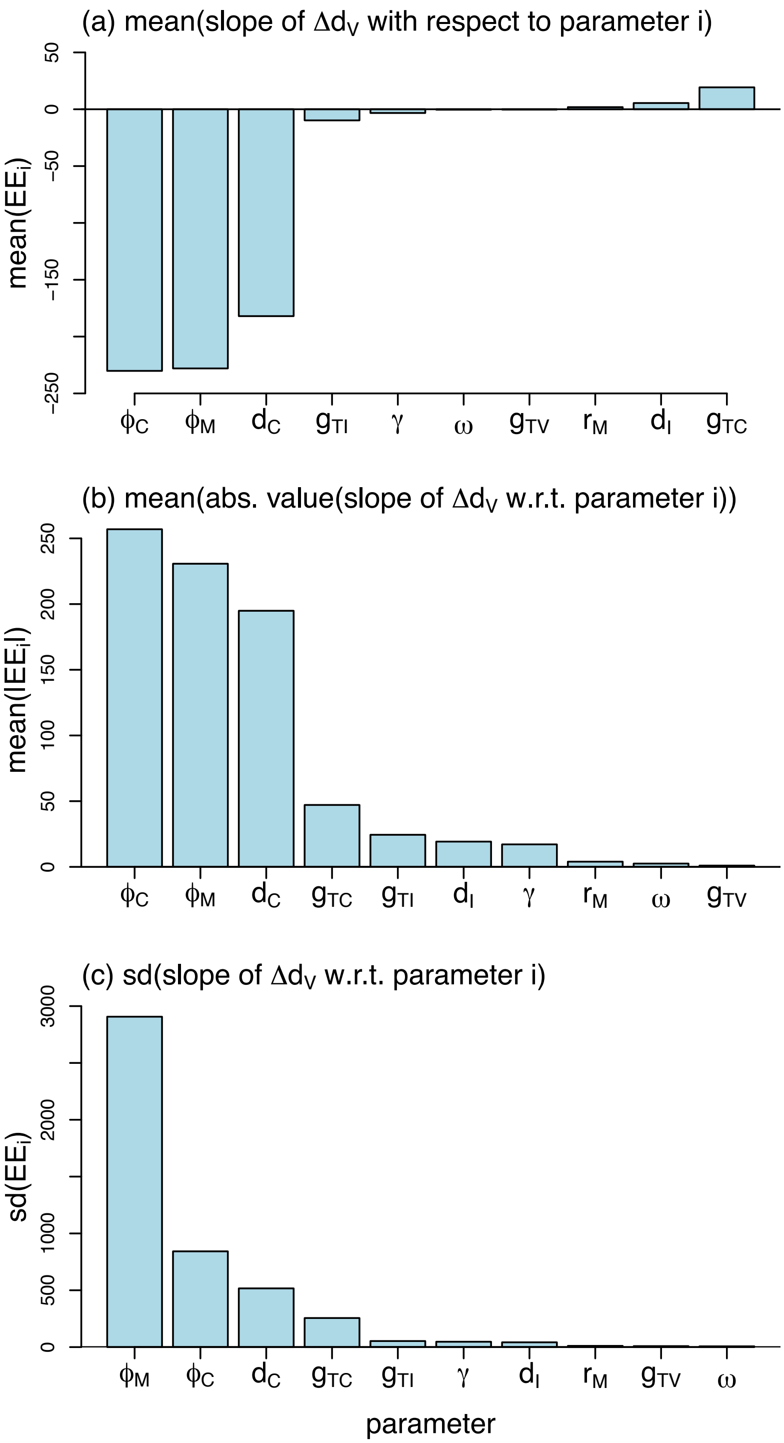

Fig A6. Local sensitivity analysis for the stage-structured macroalgae model, using EE_i, in response to a 10% one-at-a-time increase in each parameter as the measure of local sensitivity.

Shown are (a) the mean, (b) the mean of the absolute values, and (c) the standard deviation of EE_i for a sample of 1000 parameter sets drawn from the feasible ranges of parameters, subject to the constraints described in the text.

References

1. Morris MD. Factorial Sampling Plans for Preliminary Computational Experiments. Technometrics. 1991;33: 161–174. doi:10.2307/1269043

2. Wainwright HM, Finsterle S, Jung Y, Zhou Q, Birkholzer JT. Making sense of global sensitivity analyses. Comput Geosci. Elsevier; 2014;65: 84–94. doi:10.1016/j.cageo.2013.06.006

3. Saltelli A, Ratto M, Andres T, Campolongo F, Cariboni J, Gatelli D, et al. Global Sensitivity Analysis. The Primer. Global Sensitivity Analysis. The Primer. 2008. doi:10.1002/9780470725184
