# Supplementary material for: Macroalgae size refuge from herbivory promotes alternative stable states on coral reefs
Source: PLoS One. 2018 Sep 18;13(9):e0202273. doi: 10.1371/journal.pone.0202273 (PMC6143192; doi:10.1371/journal.pone.0202273)
Supplement: S3 File — (PDF) [file pone.0202273.s003.pdf]

S3 File. Details of global sensitivity analysis

To perform both local and global stability analysis, we constrained the parameters to include only combinations of parameters for which the system was coral dominated ($C > 0.4$, $M = (M_V + M_I) < 0.1*M_{high}$) for very high levels of herbivory ($d_V = 12$ y^{-1}), and macroalgae-dominated ($C < 0.1*C_{high}$, $M = (M_V + M_I) > 0.4$) for very low levels of herbivory ($d_V = 0.01$ y^{-1}). This excludes parameter combinations for which one of the two types of taxa always dominates the space on the reef regardless of the level of herbivory.

We also included only parameter values for which $0.01 < crit_M < 12$. This final constraint was added so that the full range d_V values resulting in alternative stable states (i.e. Δd_V) could be determined. This excludes the parameters in the peak at $\Delta d_V = 12$ in Fig. 1d in the main text. The reason for including this constraint is that the numerical methods that we used to calculate Δd_V required imposing an upper limit to the parameter range over which we calculated the equilibrium values. Including all of the points for which $\Delta d_V > 12$ as $\Delta d_V = 12$, rather than their actual value, would have introduced an additional source of variation into the results. This had little effect on metrics of parameter influence generated by the Random Forest method (because this method is non-parametric and does not assume linearity in the response, but had a large effect on the results of Sobol's method, which assumes linearity.

For both local and global sensitivity analysis, equilibria were calculated numerically, and confirmed through simulations using the R package rootSolve.

Global sensitivity analysis using Sobol's method

This GSA method is frequently used in computer science applications, but has only occasionally invaded the ecological literature [e.g., ,1–3], but has not been widely adopted.

We used the Sobol/Saltelli method [4,5] to calculate the sensitivity of each of our output metrics Y to each parameter i in the model. The output metrics Y included hyst (a binary variable, 1 or 0, indicating whether or not $\Delta d_V > 0$, respectively), Δd_V, $crit_C$, $crit_M$, C_{high}, and M_{high}, as defined in the main text. For each of these output metrics we calculate S_i = the first order sensitivity of that output to parameter i (Sobol's Index), and $S_{t,i}$ = the total sensitivity of that output to parameter i, for each parameter in the model. S_i describes the relative contribution of parameter i to the variance in the output metric, excluding interactions between parameter i and the other parameters in the model. $S_{t,i}$ describes the total effect of parameter i, including all interactions.

To calculate S_i and $S_{t,i}$, we followed the algorithm in Saltelli et al. [5] and Glen and Isaacs [6]. This involved creating two matrices A and B, each containing n = 10,000 random parameter combinations from the feasible ranges of parameters in Table 1 (using a uniform distribution) in the main text, subject to the additional constraints described in the main text. Matrices A and B each have dimensions n x p, where p is the number of parameters in the model, and each of the n rows in A and B is a set of random parameters. From A and B we created a matrix C_i for each parameter i. All entries in C_i are identical to those in matrix B, except for the values in the ith column, which are instead taken from matrix A.

We then calculated each of the output metrics ($hyst$, Δd_V, $crit_C$, $crit_M$, C_{high}, and M_{high}) for each of the sets of parameters in matrices A, B, and C_i. In the main text we concentrate on the sensitivity of Δd_V to the parameter values, and we will use that output metric as an example here, but this procedure was repeated to calculate the sensitivities for each of the other output metrics. The values of Δd_V for parameter sets A, B, and C_i are each vectors of length n: $\{a_m\}$, $\{b_m\}$, and $\{c_{i,m}\}$, respectively, where $m = 1, ...n$.

Following Glen and Isaacs [6] and Wainwright et al. [7], we computed S_i as the correlation coefficient between $\{a_m\}$ and $\{c_{i,m}\}$:

$$S_i = \frac{1}{\sigma_y^2}\frac{1}{(n-1)}\sum_{m=1}^{n}[(a_m - \mu_y)(c_{i,m} - \mu_y)]$$

where μ_y is the overall mean, and σ_y^2 is the overall variance, of the output metric Y (in this case Δd_V). For a given row m, parameter sets A and C_i have the same value of parameter i, and different values of all other parameters (selected at random). If parameter i has a very large influence in determining the output, then a_m and $c_{i,m}$ should be similar, and the vectors $\{a_m\}$ and $\{c_{i,m}\}$ should be correlated, leading to S_i close to 1. If parameter i has little influence on the output, then $\{a_m\}$ and $\{c_{i,m}\}$ should be uncorrelated, and S_i should be close 0. Fig A7 illustrates the meaning of the S_i measure of the importance of the parameters of the stage-structured model on Δd_V.

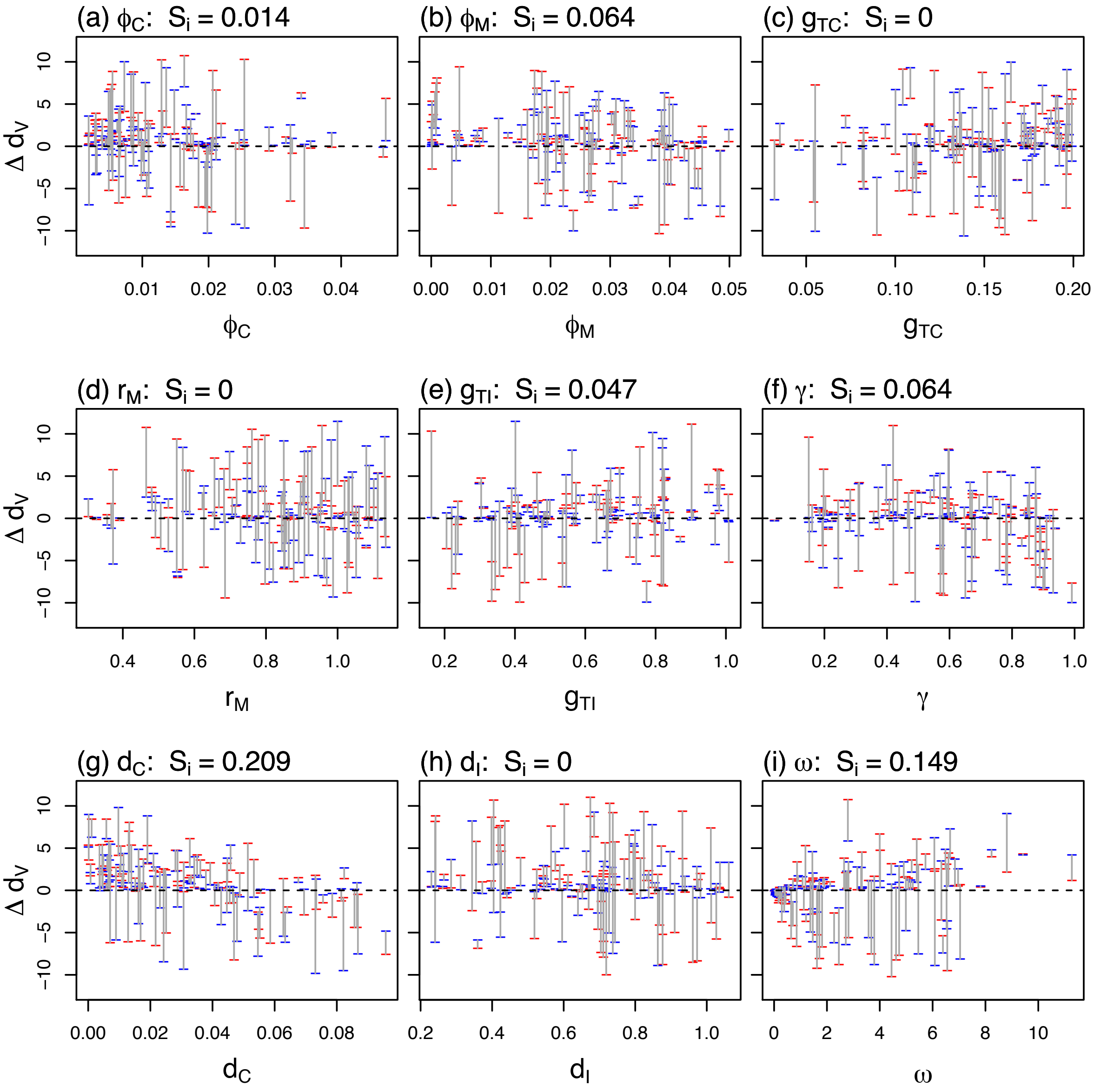

Fig A7. Illustration of global sensitivity of Δd_V to the parameters in the stage-structured model using S_i, the first-order index from Sobol's method. In each panel, the vertical gray lines connect the value of Δd_V for a parameter set in matrix A (indicated by the red points) with the value of Δd_V for the corresponding parameter set in matrix C_i (indicated by the blue points). Each pair of red and blue points shares the same value of parameter i, but differs in all other parameters. If parameter i is very influential in determining d_V, then the red and blue points will

be close together (i.e. the vertical gray lines will be short). For illustrative purposes, only 100 randomly selected combinations of parameters are shown, and the results for the non-influential parameter g_{TV} are not shown.

We computed $S_{t,i}$ as 1 - (the correlation coefficient between $\{b_m\}$ and $\{c_{i,m}\}$):

$$S_{t,i} = 1 - \frac{1}{\sigma_y^2}\frac{1}{(n-1)}\sum_{m=1}^{n}\left[(b_m - \mu_y)(c_{i,m} - \mu_y)\right]$$

For a given row m, parameter sets B and C_i have the same value of all of the parameters, except parameter i. In this case, if parameter i has a very large influence in determining the output, then b_m and $c_{i,m}$ should be different from each other (because of the effect of changing parameter i), and the vectors $\{b_m\}$ and $\{c_{i,m}\}$ should be uncorrelated (correlation coefficient close to zero). As $S_{t,i}$ is defined as 1- (the correlation coefficient between $\{b_m\}$ and $\{c_{i,m}\}$), this results in $S_{t,i}$ close to 1.

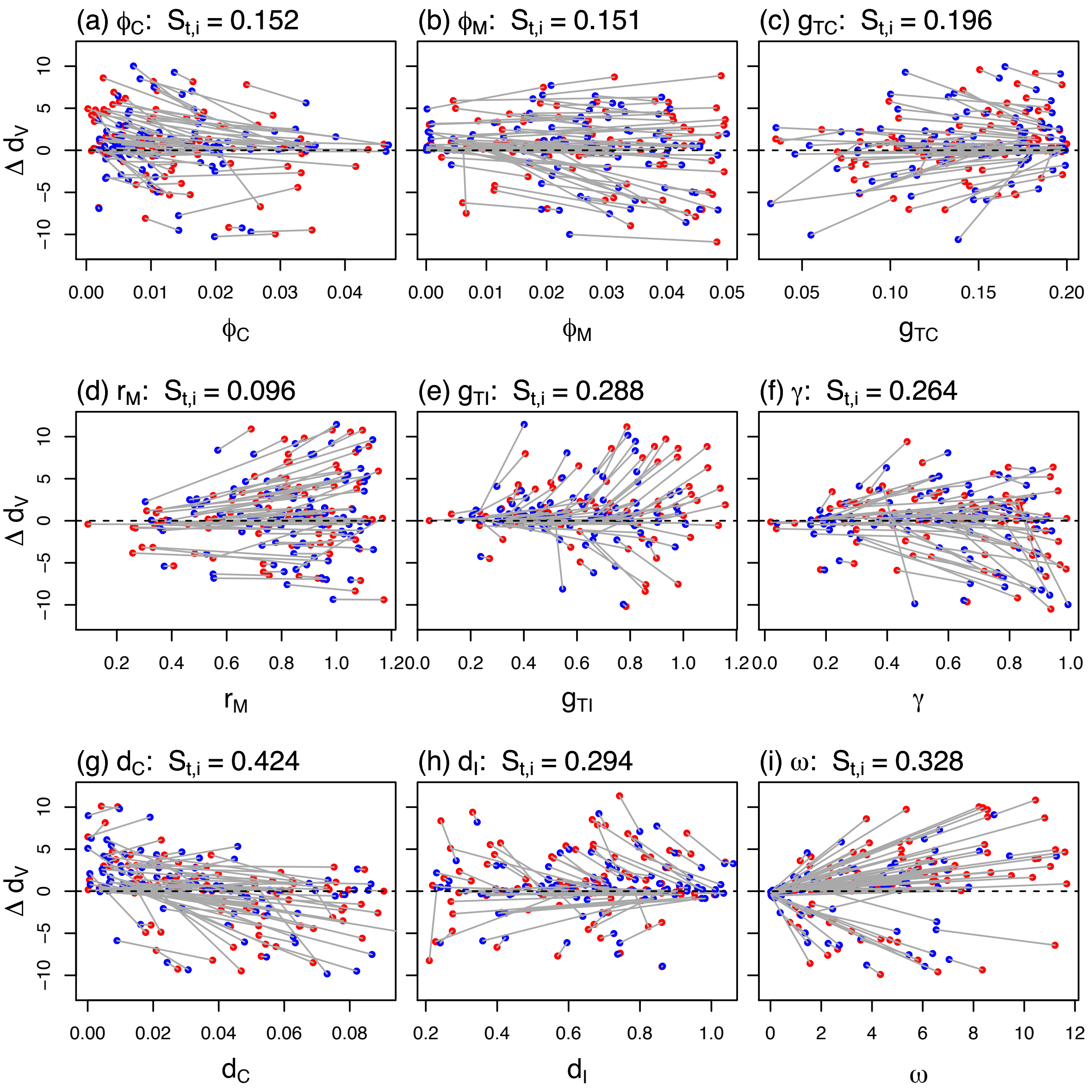

Fig A8. Illustration of global sensitivity of Δd_V to the parameters in the stage-structured model using $S_{t,i}$, the total sensitivity index from Sobol's method. In each panel, the gray lines connect the value of Δd_V for a parameter set in matrix B (indicated by the red points) with the value of Δd_V for the corresponding parameter set in matrix C_i (indicated by the blue points). Each pair of red and blue points have different values of parameter i, but share the same values of all of the other parameters. If parameter i has no influence on Δd_V, then each gray line should have a

slope of zero. Lines with non-zero slopes indicate that changing the value of parameter i has an impact on Δd_V. For illustrative purposes, only 100 randomly selected combinations of parameters are shown, and the results for the non-influential parameter g_{TV} are not shown.

Global sensitivity analysis using random forest method

This approach has been applied previously to coral reef models [8]. Classification and regression trees (CART) recursively split a dataset into two parts (nodes), based on the value of a parameter that will maximize the homogeneity of the two subsets of the dataset. For example, Fig A9 shows the regression tree to predict Δd_V in the stage-structured model. The top node contains 100% of the dataset, for which the average Δd_V is 0.57. Dividing the dataset into two parts based on whether or not d_C is less than 0.037 creates the two most homogeneous subsets. The 44% of the data with $d_C \geq 0.037$ has an average Δd_V of -1.5 and the remaining 56% of the data has an average Δd_V of 2.2. The data in each of these nodes is partitioned recursively, based on the value of the parameter that leads to the most homogeneous subsets. The Random Forest approach [9] creates many classification or regression trees based (CART) [10] on subsets of the data set and subsets of the parameters allowing for the quantification of both the classification error and the importance of the parameters, including all of the potential non-linear interactions among the parameters.

To implement the Random Forest method, we merged parameter sets A and B into the matrix X, which contains 20,000 randomly selected combinations of parameters from across the feasible range for each parameter. We also merged their associated vectors of output metrics (e.g. Δd_V), $\{a_m\}$ and $\{b_m\}$, into the vector Y. We then used the randomForest package in R to

determine the importance of each of the parameters in X on the output vector Y. We re-scaled the relative importance values for a given output metric to sum to 1.

Results from global sensitivity analysis of unstructured model

Table A1. Results of Global Sensitivity Analysis for the unstructured model: Sobol's first-order sensitivity index, S_i, showing the influence of the model parameters on all of the output metrics defined in Fig. 1a,b in the main text. *hyst* is a binary response variable indicating whether or not alternative stable states and hysteresis are possible for any values of herbivory on macroalgae. M_{high} (not shown in Fig. 1a,b) is the equilibrium cover of macroalgae in the macroalgae-dominated state, i.e., when d_V is very low. (Zeros shown in table include actual zeros, very small values rounded to zero, and any negative values.)

parameter	S_i, first-order sensitivities					
	hyst	Δd_V	$crit_C$	$crit_M$	C_{high}	M_{high}
ϕ_C	0.013	0	0.011	0.100	0.002	0
ϕ_M	0.032	0.172	0.108	0.004	0.001	0
g_{TC}	0	0.085	0.031	0.005	0.104	0
g_{TM}	0.066	0.058	0.259	0.593	0.003	0.900
γ	0.067	0.141	0.209	0.180	0.002	0.015
d_C	0.116	0.143	0.069	0.004	0.698	0

Table A2. Results of Global Sensitivity Analysis for the unstructured model: **Sobol's total sensitivity index,** $S_{t,i}$, showing the influence of the model parameters on all of the output metrics. (Zeros shown in table include actual zeros, very small values rounded to zero, and any negative values.)

**parameter**	**$S_{t,i}$, total sensitivities**					
	$hyst$	Δd_V	$crit_C$	$crit_M$	C_{high}	M_{high}
ϕ_C	0.235	0.108	0.112	0.123	0.073	0.028
ϕ_M	0.310	0.317	0.208	0.013	0	0.015
g_{TC}	0.200	0.378	0.229	0.018	0.296	0.001
g_{TM}	0.530	0.198	0.388	0.680	0	0.978
γ	0.560	0.253	0.316	0.247	0	0.081
d_C	0.666	0.380	0.198	0.019	0.903	0.257

Table A3. Results of Global Sensitivity Analysis for the unstructured model: Standardized importance measures from Random Forest models, showing the influence of the model parameters on all of the output metrics. For each output metric from each model, the importance measures are normalized to sum to 1. Symbols in parentheses indicate whether increases in the value of that parameter leads to an increase (+), decrease (-), or no change (.) in that output metric from the model, as determined by the sign of the coefficient for that parameter from a generalized linear model, and confirmed by a general additive model. Changing the value of a particular parameter resulted in either a monotonic increase or decrease in the output metrics in most cases (as indicated by a (+) or (-), respectively, in the table). However, in a few cases the response was non-linear and unimodal, with either a humped or U-shaped response. A humped shaped response is indicated by a superscript H, and a U-shaped response is indicated by a superscript U in the table, although the overall positive or negative trend is also indicated in the table.

**parameter**	**Importance Factors from Random Forest**					
	hyst	Δd_V	$crit_C$	$crit_M$	C_{high}	M_{high}
ϕ_C	0.083 (–)	0.060 (+)	0.073 (–)	0.175 (–)	0.167 (+)	0.062 (–)
ϕ_M	0.107 (–)	0.228 (–)	0.176 (+)	0.031 (0)	0 (0)	0.061 (+)
g_{TC}	0.069 (+)	0.145 (+)	0.109 (–)	0.024 (0)	0.306 (+)	0.046 (0)
g_{TM}	0.230 (+)	0.159 $(–)^{U}$	0.255 (+)	0.436 (+)	0.013 (0)	0.569 (+)

γ	0.231<br>(−)[H]	0.197<br>(−)	0.238<br>(+)	0.299<br>(+)	0<br>(0)	0.214<br>(+)
d_C	0.281<br>(−)	0.210<br>(−)	0.149<br>(+)	0.034<br>(−)	0.514<br>(−)	0.048<br>(0)

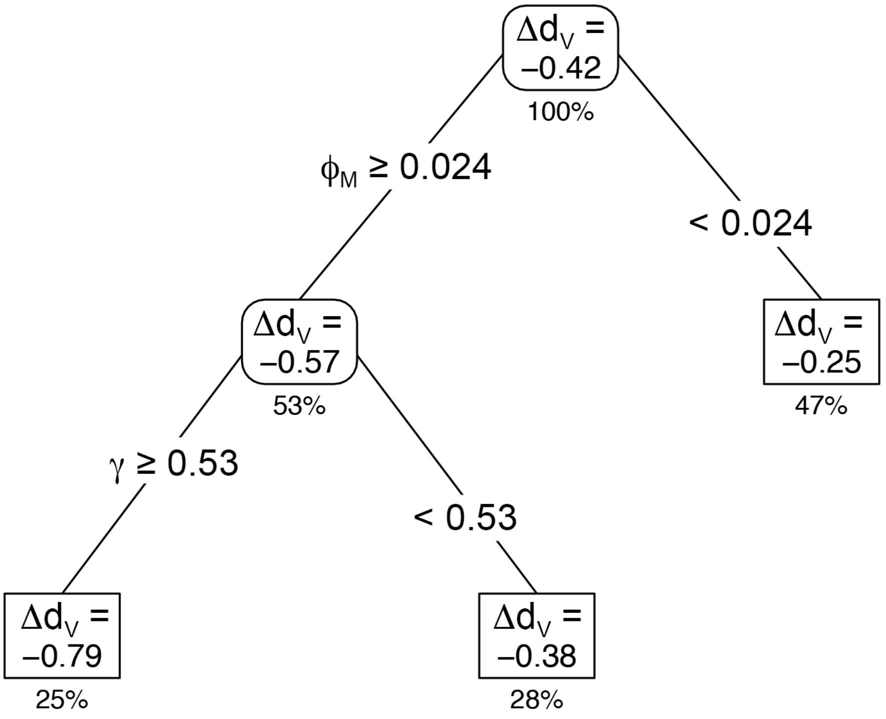

Fig A9. Pruned regression tree for unstructured model, showing the interactions among the parameters in determining Δd_V. The values inside the nodes (e.g. Δd_V = -0.42 for the top node) indicate the average value of Δd_V for the parameter points in that node. The percentage below each node indicates the % of all parameter points in that node. The criteria for parameters determining the splits are shown on each branch. Complexity parameter cp = 0.1 was used to prune the tree.

Table A4. Results of Global Sensitivity Analysis for the stage-structured macroalgae model: Sobol's first-order sensitivity index, S_i, showing the influence of the model parameters on all of the output metrics. The results for Δd_V are also shown in Fig 4c in the main text. (Zeros shown in table include actual zeros, very small values rounded to zero, and any negative values.)

parameter	S_i, first-order sensitivities					
	hyst	Δd_V	$crit_C$	$crit_M$	C_{high}	M_{high}
ϕ_C	0	0.014	0	0.005	0.003	0.008
ϕ_M	0.041	0.064	0.028	0.001	0	0.007
g_{TC}	0	0	0	0.002	0	0.007
r_M	0.001	0	0	0.035	0	0.057
g_{TV}	0.005	0.008	0	0.005	0	0.012
g_{TI}	0.031	0.047	0.008	0.030	0.003	0.037
γ	0.046	0.064	0.071	0	0	0.004
d_C	0.248	0.209	0.165	0.002	0.733	0.022
d_I	0.005	0	0	0.004	0	0.201
ω	0.216	0.149	0.065	0.428	0.003	0.627

Table A5. Results of Global Sensitivity Analysis for the stage-structured macroalgae model: Sobol's total sensitivity index, $S_{t,i}$, showing the influence of the model parameters on all of the output metrics. The results for Δd_V are also shown in Fig 4d in the main text. (Zeros shown in table include actual zeros, very small values rounded to zero, and any negative values.)

parameter	$S_{t,i}$, total sensitivities					
	$hyst$	Δd_V	$crit_C$	$crit_M$	C_{high}	M_{high}
ϕ_C	0.133	0.152	0.065	0.329	0.040	0.020
ϕ_M	0.253	0.151	0.200	0.074	0.017	0.002
g_{TC}	0.200	0.196	0.320	0.277	0.202	0.002
r_M	0.072	0.096	0.073	0.251	0.003	0.059
g_{TV}	0.023	0.003	0.001	0.004	0	0.004
g_{TI}	0.147	0.288	0.394	0.548	0.025	0.040
γ	0.217	0.264	0.397	0.402	0.052	0.012
d_C	0.572	0.424	0.691	0.266	0.831	0.003
d_I	0.130	0.294	0.418	0.525	0.022	0.166
ω	0.234	0.328	0.431	0.682	0.023	0.476

Table A6. Results of Global Sensitivity Analysis for the stage-structured macroalgae model: Standardized importance measures from Random Forest models, showing the influence of the model parameters on all of the output metrics. (The results for Δd_V are also shown in Figs 4c and 4d in the main text.) For each output metric from each model, the importance measures are normalized to sum to 1. Symbols in parentheses indicate whether increases in the value of that parameter leads to an increase (+), decrease (-), or no change (.) in that output metric from the model, as determined by the sign of the coefficient for that parameter from a generalized linear model, and confirmed by a general additive model. Changing the value of a particular parameter resulted in either a monotonic increase or decrease in the output metrics in most cases (as indicated by a (+) or (-), respectively, in the table). However, in a few cases the response was non-linear and unimodal, with either a humped or U-shaped response. A humped shaped response is indicated by a superscript H, and a U-shaped response is indicated by a superscript U in the table, although the overall positive or negative trend is also indicated in the table.

**parameter**	**Importance Factors from Random Forest**					
	$hyst$	Δd_V	$crit_C$	$crit_M$	C_{high}	M_{high}
ϕ_C	0.079 (−)	0.095 (−)	0.061 (−)	0.098 (−)	0.091 (+)	0.042 (−)
ϕ_M	0.116 (−)	0.117 (−)	0.100 (+)	0.036 (−)	0.035 (−)	0.006 (0)
g_{TC}	0.079 (+)	0.071 (+)	0.091 (−)	0.044 (−)	0.221 (+)	0.030 (0)
r_M	0.043	0.040	0.026	0.041	0.036	0.132

	(+)	(+)	(+)	(+)	(0)	(+)
g_{TV}	0.006<br>(0)	0.006<br>(0)	0.007<br>(0)	0.006<br>(0)	0.019<br>(0)	0.020<br>(0)
g_{TI}	0.076<br>(+)	0.075<br>(+)	0.057<br>(+)	0.134<br>(+)	0.032<br>(−)	0.054<br>(+)
γ	0.111<br>(0)^{H}	0.113<br>(−)^{H}	0.103<br>(+)	0.096<br>(+)	0.042<br>(−)	0.061<br>(0)
d_C	0.254<br>(−)	0.212<br>(−)	0.269<br>(+)	0.098<br>(0)	0. 448<br>(−)	0.033<br>(0)
d_I	0.057<br>(0)^{H}	0.077<br>(+)^{H}	0.088<br>(−)	0.095<br>(−)	0.031<br>(+)	0.169<br>(−)
ω	0.178<br>(+)	0.195<br>(+)^{U}	0.198<br>(+)	0.350<br>(+)	0.046<br>(−)	0.454<br>(+)

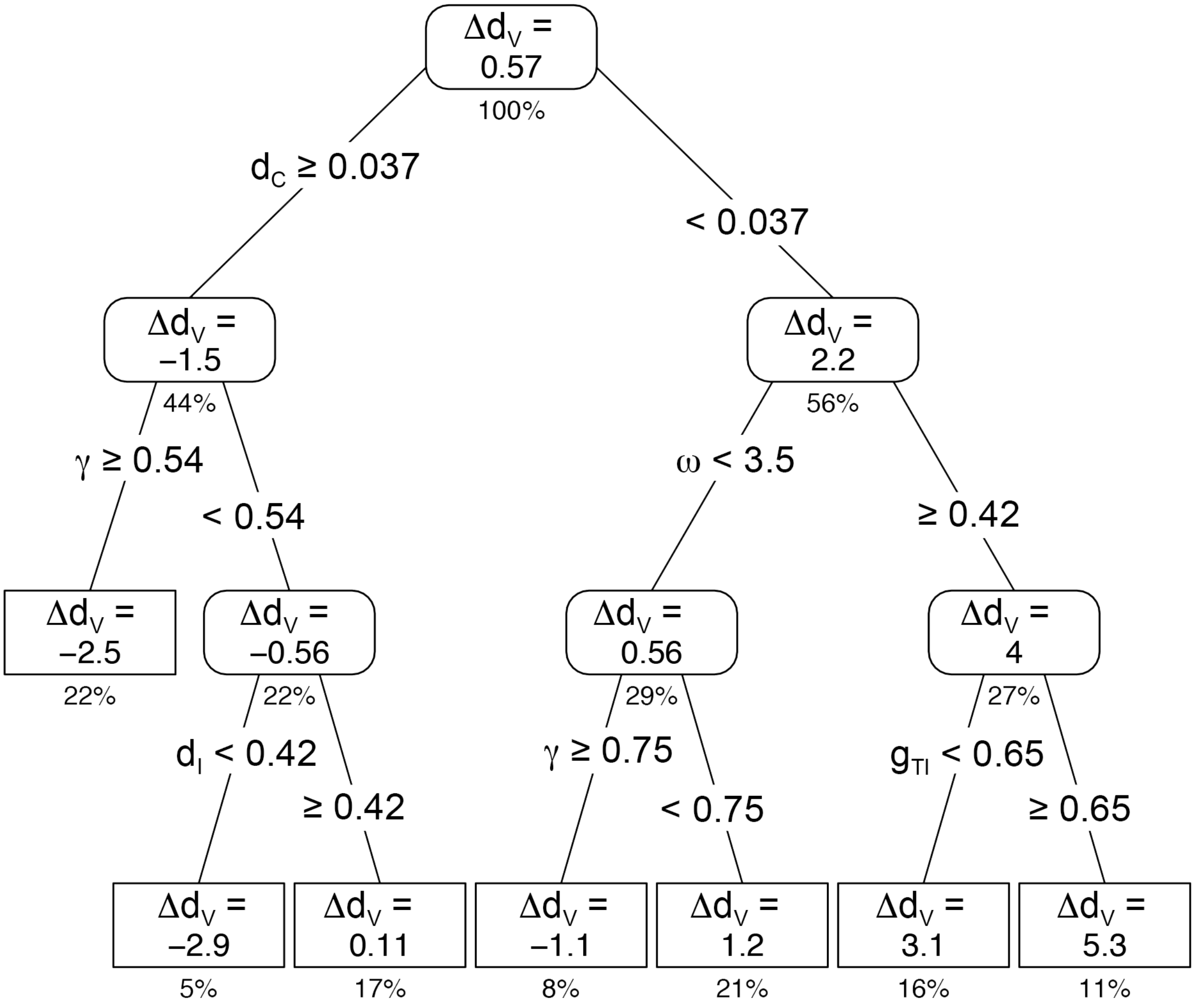

Fig. A10. Pruned regression tree for stage-structured macroalgae model, showing the interactions among the parameters in determining Δd_V. The values inside the nodes (e.g. Δd_V = 0.57 for the top node) indicate the average value of Δd_V for the parameter points in that node. The percentage below each node indicates the % of all parameter points in that node. The criteria for parameters determining the splits are shown on each branch. Complexity parameter *cp* = 0.015 was used to prune the tree.

References

1. Ellner S, Fieberg J. Using PVA for management despite uncertainty: effects of habitat, hatcheries, and harvest on salmon. Ecology. 2003;84: 1359–1369. Available: http://www.esajournals.org/doi/abs/10.1890/0012-9658(2003)084%5B1359:UPFMDU%5D2.0.CO%3B2
2. Perz SG, Muñoz-Carpena R, Kiker G, Holt RD. Evaluating ecological resilience with global sensitivity and uncertainty analysis. Ecol Modell. 2013;263: 174–186. doi:10.1016/j.ecolmodel.2013.04.024
3. Dunn JM, Davis S, Stacey A, Diuk-Wasser MA. A simple model for the establishment of tick-borne pathogens of Ixodes scapularis: A global sensitivity analysis of R0. J Theor Biol. Elsevier; 2013;335: 213–21. doi:10.1016/j.jtbi.2013.06.035
4. Sobol IM. Global sensitivity indices for nonlinear mathematical models and their Monte Carlo estimates. Math Comput Simul. 2001;55: 271–280. doi:10.1016/S0378-4754(00)00270-6
5. Saltelli A, Ratto M, Andres T, Campolongo F, Cariboni J, Gatelli D, et al. Global Sensitivity Analysis. The Primer. Global Sensitivity Analysis. The Primer. 2008. doi:10.1002/9780470725184
6. Glen G, Isaacs K. Estimating Sobol sensitivity indices using correlations. Environ Model Softw. Elsevier Ltd; 2012;37: 157–166. doi:10.1016/j.envsoft.2012.03.014
7. Wainwright HM, Finsterle S, Jung Y, Zhou Q, Birkholzer JT. Making sense of global sensitivity analyses. Comput Geosci. Elsevier; 2014;65: 84–94. doi:10.1016/j.cageo.2013.06.006
8. Edmunds PJ, Adjeroud M, Baskett ML, Baums IB, Budd AF, Carpenter RC, et al.

Persistence and change in community composition of reef corals through present, past, and future climates. PLoS One. 2014;9. doi:10.1371/journal.pone.0107525

9. Breiman L. Random forests. Mach Learn. 2001;45: 5–32. doi:10.1023/A:1010933404324

10. Breiman L, Friedman JH, Olshen RA, Stone CJ. Classification and Regression Trees. Chapman and Hall/CRC. 1984.
